# Weight Bias 2.0: The Effect of Perceived Weight Change on Performance Evaluation and the Moderating Role of Anti-fat Bias

**DOI:** 10.3389/fpsyg.2021.679802

**Published:** 2021-07-16

**Authors:** Yueting Ji, Qianyao Huang, Haiyang Liu, Caleb Phillips

**Affiliations:** ^1^Business School, Central University of Finance and Economics, Beijing, China; ^2^Guanghua School of Management, Peking University, Beijing, China; ^3^Department of Management, London School of Economics and Political Science, London, United Kingdom

**Keywords:** weight bias, weight change, anti-fat bias, phase-shifting perspective, performance evaluation

## Abstract

Overweight employees are viewed as lazy, slow, inactive, and even incapable. Even if such attributes are false, this perspective can seriously undermine others' evaluation of their work performance. The current study explores a broader phenomenon of weight bias that has an effect on weight change. In a longitudinal study with a time lag of 6 months, we surveyed 226 supervisor-employee dyads. We found supervisor perceptions of employee weight change notably altered their evaluation of the employee performance from Time 1, especially following low vs. high Time-1 performance evaluation. Meanwhile, the moderating effects among different levels of supervisor anti-fat bias functioned as boundary conditions for such performance evaluation alteration. In particular, the interaction between the Time-1 performance evaluation and the impact of supervisor perception of employee weight change on the Time-2 performance evaluation was significant only if supervisors held a stronger anti-fat bias.

## Introduction

Bias seems ubiquitous in the workplace (Rattan and Dweck, 2018). Each employee's negatively stereotyped characteristics, such as sexuality, ethnicity, skin color, age, disability, and even body weight, can incite mistreatment (Dovidio, 2010). Despite the difficulty inherent in overcoming bias, evidence in the last decade has indicated positive and encouraging progress in alleviating nearly all forms of bias (Colella et al., 2017). Nonetheless, progress in one of the most pervasive forms of bias, namely, weight bias, appears to be stalled (Täuber et al., 2018). For instance, a recent study involving 4.4 million implicit association tests (IATs) from online U.S. participants over 10 years (from 2007 to 2016) revealed that implicit attitudes on sexuality, ethnicity, skin color, age, and disability had shifted toward neutrality; however, the weight bias had shifted in a negative manner (Charlesworth and Banaji, 2019). Indeed, abundant anecdotal evidence confirms the continued existence of workplace weight bias. Such bias is usually designated with various terms in the world media, such as “fat chance” in the *Harvard Business Review* (Fryer and Kirby, 2005), “fat shaming” in the *New York Times* (Kolata, 2016), “obesity discrimination” in the *BBC News* (Szrodecki, 2018), “size ceiling” in the *Guardian* (Van der Zee, 2017), and “weight-ism” in the *ABC News* (Dye, 2008).

Despite the increasing likelihood of experiencing weight-based discrimination, people are undeniably becoming progressively heavier. Statistics show that the obesity and overweight rates in U.S. adults with income of more than $15,000 per year are 32.63 and 35.10%, respectively and the situation will become worse in the coming decade (Center for Disease Control Prevention, 2019). Overweight working adults are more likely to be victims of weight discrimination and bias partly because of a lack of weight-related anti-discrimination legislation. For example, only one U.S. state prohibits discrimination based on weight^1^. Justifying the need for such anti-discrimination policy, Sassi (2010) found that obese employees are paid 10 percent less than non-obese employees, even whilst carrying out the same tasks and holding equivalent positions. As such, the scientific view toward workplace weight bias and discrimination is necessary and urgent. However, organizational research on weight bias has lagged far behind. For instance, recent meta-analysis studies on different forms of workplace bias found the research attention on weight bias in traditional organizational journals is quite limited (Dhanani et al., 2018). Unsurprisingly, Brownell et al. (2005) also argued that organizational research on weight bias is considerably underdeveloped given the striking prevalence of weight bias and the severity of its consequences.

The current study aims to direct increasing attention toward and promote understanding of weight-related bias in the workplace. We posit that workplace weight bias may be considerably more rampant than the current research would suggest. In particular, the targets of weight bias may not be limited to individuals who are presently overweight. Those who have altered their weight can also be “victims” of such bias. Because one's body weight can change (Thomas et al., 2011), weight bias should encompass the effect on weight change. Nevertheless, weight bias remains primarily operationalized as the significant main effect of an individual's current weight on how others evaluate their qualifications or job performance when all other qualities are controlled (Rudolph et al., 2009). This prevalent fixed-characteristic paradigm for examining workplace weight bias seems to have largely neglected the changing state of one's weight (i.e., weight loss, maintaining the same weight, and weight gain), and such a situation may lead to an underestimation of the scope and consequence of weight bias in the organizational view (King et al., 2005; Levine and Schweitzer, 2015). In particular, research has established that more recent information may take a more important role in predicting one's evaluations than older information (Sharif and Oppenheimer, 2016). As such, weight change may transcend weight in forming supervisors' evaluations. However, previous research seems to have largely neglected such possibilities, which may lead to incomplete conclusions about weight bias.

Indeed, an individual's weight is never fixed. Research revealed that employees' BMI rankings could change rapidly (from underweight, normal weight, overweight, obesity, to extreme obesity; WHO, 2004) in as short a window as several months (Bhutani et al., 2017). However, the theoretical foundation of the effect of weight change on biased evaluation remains inchoate because of limited research attention. Such scarcity arises as previous research on bias and discrimination exclusively focused on fixed characteristics and rarely considered the changeable ones (Howard, 2008; Granberg, 2011). Many major forms of bias are attached to characteristics that are recognized as innate and usually permanent (e.g., race, gender, and skin tone). Therefore, some changeable characteristics may be regarded as less relevant to their constant counterparts. This situation likely contributes to the lack of attention to this topic. Nevertheless, certain forms of bias are directed toward characteristics that may not be fixed (e.g., alcohol addiction and being overweight). If the characteristic targeted by bias is changeable, individuals may aggravate or alleviate the bias, thereby changing others' evaluation of them (Biernacki, 1986). Being overweight is such a typical “changeable” characteristic (Blaine et al., 2002). Accordingly, an individual's weight change is highly likely to affect others' perception, cognition, and especially evaluation in terms of weight bias. But the perspective that facilitates our understanding of why and when the employee (observee) actual weight change can affect supervisor (observer) evaluation of them due to weight bias is still missing.

## Weight Change and Weight Bias: A Phase-Shifting Perspective

A systematic understanding of the role of weight change in workplace weight bias (i.e., in biasing performance evaluation) begs several questions. Will an individual's performance evaluation, which may presently be unaffected by weight bias, be subsequently undermined following weight gain? Will an individuals' performance evaluation, which is currently subject to weight bias, subsequently improve due to weight loss? To answer these questions, this study adopts a phase-shifting perspective (Soenen et al., 2017; Stouten et al., 2018).

Generated from heuristic theories (Proudfoot and Lind, 2015; Audrey Korsgaard et al., 2018), the phase-shifting perspective posits that an alteration of a previous evaluation condition can induce observers to reevaluate the new condition and modify their previous evaluation (Lind, 2001). A new evaluation remains relatively stable until another change occurs (Proudfoot and Lind, 2015). However, when any such change happens, observers must perceive the change before they can shift their evaluation. Such perception is a prerequisite for the commencement of a new evaluation phase, during which a prior evaluation is likely to be modified and observers process the change of the condition to generate a novel evaluation or modify the previous one (Jones and Skarlicki, 2013). Because different individuals can uniquely process the same change (Gawronski and Bodenhausen, 2006; Audrey Korsgaard et al., 2018), such change-related information-processing may lead to remarkably distinct evaluation alteration consequences. Following such logic, employee (observee) actual weight change may not necessarily alter supervisor (observer) performance evaluations, whereas supervisor perception of whether a weight change has transpired would cause such modifications. That is, to alter supervisor performance evaluations, their perception of weight change should be more proximal than actual weight change. Meanwhile, the supervisors' individual differences that shape how they process such weight change should also be considered an important moderation mechanism.

In particular, this perspective offers two advantages. First, it highlights the importance of *perception* (Lind, 2001). In particular, an actual change requires the observers to perceive it before it can influence them (Soenen et al., 2017). Lind (2001) proposed that the perception of change is defined as the apprehension of a certain change that can trigger one to reevaluate circumstances. Pashler (1988) described the perception of change as individuals detecting whether a change has occurred. Similarly, Audrey Korsgaard et al. (2018) explained the perception of change as an individual's sense of a change transpiring. When applied to the weight-change context, we argue that perception of change refers to whether the observer thinks the observees changed their weight. Such perception should contain three possibilities, namely, perceiving a weight loss, a weight gain, and no change. Each possibility may lead to different reactions from the observer. Levine and Schweitzer (2015) suggested weight loss is associated with an increased evaluation of competence and self-discipline. By contrast, Jackson et al. (2014) argued that individuals experience more discrimination and stigmatization after gaining weight. Thus, the perception of weight change should be more proximal than the actual weight change in driving the observers' reevaluation process.

Second, the phase-shifting perception also emphasizes the crucial role of the observers' reaction to the change. When a change occurs, individuals process change-related information to create or revise a previous evaluation (Lind, 2001). One's pre-dispositioned bias toward/against the change can play an important role during such information-processing. Specifically, pre-dispositioned bias can crucially affect information-processing, because people typically prefer information that supports their bias to information that challenges it (Hart et al., 2009). Taber and Lodge (2006) found that when individuals holding a specific bias encounter bias-inconsistent information, they try to discredit such information. In the case of weight change, different individuals can have different interpretations of weight-change information due to their prior bias regarding overweight people (i.e., anti-fat bias; Schwartz et al., 2006). For example, people who think weight loss after being overweight indicates self-discipline and self-control may believe weight loss means a change in one's attributes as well (Blaine et al., 2002). Consequently, they will revise (alleviate) their previously biased evaluation. Other individuals may think being overweight is unrelated to one's attributes, and they will regard a weight change as irrelevant information and their evaluation will remain the same. Therefore, we examine whether anti-fat bias can influence supervisors' reaction to employee weight change when making performance evaluations.

By drawing upon this phase-shifting perspective of weight change, we offer an overarching framework for understanding and predicting supervisors' response to employee weight change (i.e., weight loss, weight gain, or no change) in terms of performance evaluation. As such, this study contributes to the literature in three ways. First, we enhance the weight-bias research by introducing the analysis of weight change into the current fixed-characteristic research paradigm. By following the phase-shifting perspective (Lind, 2001; Proudfoot and Lind, 2015), we are among the first to identify the important role of weight change in altering others' evaluation. Meanwhile, we find that supervisor perception of employee weight change is more proximal than the actual employee weight change in shaping the supervisor evaluation alteration of employee performance. Second, by considering the moderating role of supervisor anti-fat bias in determining the consequences of their perception of employee weight change, this work also contributes to the phase-shifting perception, such that we explore how individuals' biases can affect the evaluation process. Third, our research also adds to the performance-evaluation literature by identifying the factors that lead to performance evaluation alterations, or, in another sense, moderate the link between performance evaluations from two different time points. This work establishes the role of employee physical change between the two time points. In particular, employee weight change (or, in a broader sense, their appearance change) is a possible conditional change that affects performance evaluation alteration. That finding may be explored further in future performance evaluation research.

## Argument and Hypothesis

### Effect of Perception of Weight Change on Performance-Evaluation Alteration

Performance evaluation is one of the most important workplace outcomes that may be influenced by weight. Bernardin et al. (2016) suggested performance evaluations are particularly susceptible to the effects of stereotypes and bias (see also Moers, 2005). Advancing this line of research, we first posit that supervisor perception of employee weight change may moderate the relationship between prior-change (Time 1) and post-change (Time 2) performance evaluations. When supervisors do not perceive an employee weight change (even if such change occurred), the original and later performance evaluations should largely remain the same. Therefore, prior-change performance evaluation (Time 1) should be associated with the subsequent (Time 2) evaluation. The situation may differ if supervisors perceive a weight change. In this case, supervisors consider the information brought about by the weight change, and can potentially update their Time-1 performance evaluation, tempering the relationship between the Time-1 and Time-2 evaluations.

We argue such potentiality is determined by the supervisor existing evaluation of employee Time-1 performance. That is, the evaluations of low-performance employees in Time 1 are more likely to be affected by a weight change, as previous studies indicate that clearly excellent qualifications and performance evaluation may overcome the negative bias that appears to be frequently associated with weight (McKee and Smouse, 1983; Klesges et al., 1990). In the same vein, Heilman et al. (1997) also found that clear information about prior successful performance may overcome a certain degree of bias in evaluation-making. Nieminen et al. (2013) proposed that positive past performance activates the process of individuation; hence, specific information (i.e., past performance information) may lead the observer to view an overweight employee in a non-stereotyped manner. However, employees with inferior qualifications and performance are more likely to experience bias if they are overweight. Gaertner and Dovidio (2000) found evidence of discrimination against job applicants with marginal credentials but not against those with clearly strong credentials. Aversive theory holds (Gaertner and Dovidio, 1986) that low performance or qualifications can aggravate the negative effect of weight bias, because a negative performance history can reinforce observers' biased evaluations of overweight ratees (Steiner and Rain, 1989; Salvemini et al., 1993). Thus, even when a supervisor perceives an employee's weight change, that employee Time-1 performance evaluation should also play a role in determining how the supervisor updates the Time-2 performance evaluation; that is, Time-2 performance evaluation is more likely to be revised for employees with low Time-1 performance evaluation. Conversely, employees with high Time-1 performance evaluation can be immune or less alert to the new weight-change information.

In addition to the determining role of Time-1 performance evaluation, the moderation effect of weight loss vs. weight gain change perception can be different. Such a difference should be examined because weight loss and weight gain signal different employee attributes, thereby leading to dissimilar evaluative consequences (Jackson et al., 2014; Levine and Schweitzer, 2015). For instance, losing weight usually signals self-control, re-established competence, and improved popularity and ability (Fardouly and Vartanian, 2012). On the other hand, gaining weight signals laziness, a lack of self-discipline, unattractiveness, social ineptness, lack of cooperation, and low intellect (Madey and Ondrus, 1999). Thus, according to the phase-shifting perspective (Lind, 2001), supervisors who perceive a weight change will consider the new information generated by the change when revising their previous evaluations. Given that weight gain and weight loss produce different information, the way in which the supervisors revise their previous evaluations should also differ.

Specifically, if supervisors perceive employee weight loss, they may believe such employees have improved in terms of self-control, competence, and ability (Fardouly and Vartanian, 2012). These attributes are especially effective for employees with low Time-1 performance evaluation. Accordingly, low Time-1 performance evaluation can be alleviated by Time 2 due to the positive information brought about by weight loss. However, for employees with high Time-1 performance evaluation, the beneficial information from weight loss will be less effective, because high-performer evaluation is more immune to weight bias (Gaertner and Dovidio, 2000; Nieminen et al., 2013). Therefore, the association between Time-1 and Time-2 performance evaluations is weakened in cases of weight loss compared to cases with no new weight-change perception.

Conversely, if supervisors perceive employee weight gain, the consequence can be dissimilar. Not only are employees with low Time-1 performance evaluation more vulnerable to weight bias (Nieminen et al., 2013), but their past low-performance evaluation information can be used as justification for their supervisors' weight bias (Salvemini et al., 1993). As employees gain more weight, supervisors can regard them as less motivated (Larkin and Pines, 1979) and less competent (Levine and Schweitzer, 2015). On the one hand, if supervisors believe that weight gain employees lack motivation, which directs particular behavior toward achieving a specific goal (Sansone and Harackiewicz, 2000), then they are likely to believe that weight gain employees would perform worse in their work (Taghipour and Dejban, 2013). On the other hand, competence-related negative judgments are likely to prevent supervisors from giving credits to these employees (Bento et al., 2012). That is, supervisors are likely to undermine these employees' contributions and overstate their responsibility for failures because of the perceived competence change. Thus, supervisors can punish them with even lower performance evaluations. Weight-gain information for employees with high Time-1 performance evaluation can be less relevant, because their past positive performance information can make supervisors evaluate them in a non-stereotyped manner (Nieminen et al., 2013). Their high Time-1 performance evaluation may stay similar because only weight-based information (which is considered less relevant) is introduced at Time 2. In sum, low Time-1 performance evaluation may lead to an even lower Time-2 performance evaluation, and a high Time-1 performance evaluation may be retained at Time 2. Therefore, the association between Time-1 and Time-2 performance evaluations may be enhanced in cases with weight gain compared with situations in which supervisors perceive no weight change. Given such evidence, we propose the following hypothesis.

1. ***Hypothesis 1:****Supervisor perception of employee weight change moderates the positive relationship between Time-1 and Time-2 performance evaluations to these employees. Specifically, supervisor perception of weight gain would enhance the positive relationship, whereas perception of weight loss would mitigate it*.

We also hypothesize a relationship between employee actual weight change and supervisor perception of it. Such a relationship is important in relation to the phase-shifting perspective (Lind, 2001), because people can be blind to seemingly obvious changes (for reviews, see Simons and Rensink, 2005). The change size or magnitude is a commonly examined factor that influences whether one can perceive an actual change (Stolz and Jolicoeur, 2004; Vierck and Kiesel, 2008). In a similar vein, Strack and Deutsch (2004) argued that change intensity plays a major role in the activation of the perception of a change. In our case, the magnitude of employee weight change, or more precisely, the body type change, should be the key to trigger their supervisor perception of the weight change. That is, the actual and perceived weight change should be significantly related to each other.

To better capture the body type change, we introduced the concept of BMI for two reasons. First, BMI, which was brought up by Belgian scientist Lambert Adolphe Jacques Quetelet, is the most commonly used measure of body type (Judge and Cable, 2011), and prior research has proved the relationship between each BMI category and different body types (e.g., Bulik et al., 2001). Second, BMI is an algebraic combination of height and weight. Height is necessary to take into account, since the effects of weight change on varying body types fundamentally depends on height. Therefore, BMI change is more observable and comparable between persons than actual weight change. Thus, we present the following hypothesis.

1. ***Hypothesis 2:****Employee weight change, measured by BMI change, from Time 1 to Time 2 is significantly related to their supervisor perception of their weight change at Time 2*.

### Anti-fat Bias as an Important Boundary Condition

Although weight bias seems pervasive and universal, some individuals can have less negative attitudes and stereotypes toward overweight persons and do not think of being overweight as a disadvantage. Anti-fat bias is the variable that measures such attitude and cognition. Schwartz et al. (2006) defined anti-fat bias as the negative attitude or stereotype one holds for overweight persons, such as considering them lazy, unmotivated, and less preferable (see also, Agerström and Rooth, 2011; Fontana et al., 2017). Research has consistently demonstrated that anti-fat bias can vary widely among individuals (Devine, 1989; Newheiser and Dovidio, 2012), such that people with a lower level of weight bias should be less negative against overweight persons and give them a less negative evaluation (Merritt et al., 2018). Accordingly, we propose that the level of weight bias supervisors hold can moderate their undermining in evaluating overweight employees' performance.

Literature on individual differences regarding weight bias presents strong empirical and theoretical support for the moderating role of anti-fat bias (e.g., Roehling et al., 2013). Devine (1989) found that participants with a strong anti-fat bias provide lower evaluations of targets with a characteristic that matches the bias, whereas participants who do not have such a bias do not provide biased evaluations of the same targets. Similarly, Agerström and Rooth (2011) demonstrated that only managers with anti-fat bias are less likely to invite an overweight job applicant for an interview; however, the case differs for a normal-weight counterpart. In terms of performance evaluation, Rudolph et al. (2012) experimentally confirmed that observers without anti-fat bias do not give lower performance evaluations to overweight ratees.

We, therefore, propose that the moderating role of the supervisor perception of employee weight change varies across supervisors with different levels of anti-fat bias. We base our logic on the speculation that supervisor anti-fat bias can shape their reactions to employee weight change. When individuals hold strong anti-fat bias, they are more likely to assign negative attributes (e.g., incompetence, emotionality, and self-indulgence) to overweight employees (Silverstein et al., 1986; Agerström and Rooth, 2011). Naturally, when perceiving employee weight change, these supervisors with strong anti-fat bias may be more likely to react to weight change, thereby resulting in performance-evaluation alteration from Time 1 to Time 2. By contrast, supervisors with low anti-fat bias can consider employee weight change as less relevant when perceiving such change, because they do not believe weight is related to one's characteristics (Rudolph et al., 2012), thereby resulting in no additional performance-evaluation alteration from Time 1 to Time 2. In other words, the perception of employee weight change may be more influential for employee performance— evaluation change when their supervisors have strong anti-fat bias. Consequently, we propose the following hypothesis.

1. ***Hypothesis 3:****Supervisor anti-fat bias moderates the interaction between their perception of employee weight change and their Time-1 performance evaluation of such employees on Time 2 performance evaluation, such that the moderating effect should be stronger when the supervisors hold a stronger anti-fat bias*.

## Research Context

### Sample and Procedure

We conducted a two-wave survey study with supervisor-employee dyads over 6 months to test our hypotheses. Participants were from three Chinese manufacturing organizations in the southeast. The human resource (HR) departments in these organizations helped recruit participants, through which we contacted 266 supervisor-employee dyads for the study. We conducted paper-and-pencil surveys twice with each participant within an interval of 6 months. In the Time-1 surveys, employees were asked to report their weight, height, and demographics. Given the relatively sensitive information, we gained participants' consent prior to sending the survey. We carefully explained the research nature, purpose, and liability issues to each participant, and guaranteed them that the survey would be transformed into data without any personal information. To avoid the “good-subject effect” (Nichols and Maner, 2008), we intentionally misstated our research purpose as a study of weight change during job transition. At the same time, we asked the supervisors to complete the Time-1 supervisor surveys, in which they reported their performance evaluation of the employee, their anti-fat bias, and demographics. Six months later, both the Time-2 employee surveys and Time-2 supervisor surveys were conducted. Employees were asked to report their weight again and supervisors provided their new performance evaluation of the employees and their perception of the employee weight change.

Our final sample consisted of 226 employee-supervisor dyads. For employees, the average age was 23.99 years old (*SD* = 2.95), 81% were male, and the average years of education were 16.23 years (*SD* = 1.82). For supervisors, the average age was 33.73 years old (*SD* = 7.96), 88% were male, and the average years of education were 15.84 years (*SD* = 1.72).

### Measures

#### Supervisor Anti-fat Bias (Time 1)

We adopted Schwartz et al.'s (2006) three-item scale to measure the supervisors' explicit attitudes and stereotyping toward being overweight. The three items, rated on a 7-point scale (1 = *totally disagree*, 7 = *totally agree*), were “I strongly prefer thin people to fat people” (attitude), “I strongly believe that thin people are more motivated than fat people” (stereotype), and “I strongly believe that fat people are lazier than thin people” (stereotype). The reliability score for this scale was 0.92.

#### Employee BMI Change (Time 1 and 2)

As suggested by previous research, utilizing BMI rather than weight alone provides a more comprehensive meaning for the concept of “weight” and a way to compare different individuals' body sizes (WHO, 2004). While the BMI calculation is not a perfect measurement and carries overestimation of body fat, it has been established as a simple, non-invasive indicator of weight measures in most people (Renehan et al., 2008; Chen et al., 2010). As such, in this study, we adopted BMI change to indicate actual weight change. BMI was calculated based on each employee's weight (W) and height (H): BMI = $\frac{W}{H^{2}}$ kg/m^2^ (Renehan et al., 2008)^2^. At Time 1, employees were asked to report their weight and height. At Time 2, employees were again asked to report their weight. A higher value indicates a bigger increase in weight^3^.

#### Supervisor Perception of Employee Weight Change (Time 2)

Conceptually, Lind's (2001) description of perceptions of change reflects discrete states, because the observer either does or does not perceive the change. This description is consistent with our definition of the perception of weight change containing three states, namely, perception of weight gain, no weight change, or weight loss. In previous change-detection research, participants were simply asked whether change occurred (e.g., Pashler, 1988; Tovey and Herdman, 2014; Soenen et al., 2017). Following this line of research, we treated the three weight-changing states as discrete. Supervisors were directly asked, “Do you think the employee you supervised has changed his or her weight significantly or not within the last 6 months?” We coded weight gain as −1, no weight change as 0, and weight loss as 1. Such a coding method was based on our hypotheses that a moderating-effect ranking exists from negative effect (weight gain), to no effect (no weight change), and to positive effect (weight loss).

#### Performance Evaluation (Times 1 and 2)

Supervisors rated each employee's performance at Time 1 and Time 2 (6 months later) using Farh and Cheng's (1997) four-item scale of task performance. We selected this scale for its wide adoption in Chinese employee samples (e.g., Brockner et al., 2001; Law et al., 2004; Chen and Aryee, 2007; Gong et al., 2009; Chen et al., 2013; Carter and Mossholder, 2015; Schaubroeck et al., 2017). Farh and Cheng (1997) verified the criterion validity of this scale by performing a regression of supervisor-rated employee performance on employee objective sale performance (correlation coefficient = 0.38, *p* < 0.01)^4^. The discriminant validity of this scale has also been verified by previous research, suggesting the performance evaluation measure by the four-item scale is distinct from related constructs, such as supervisor-rated job dedication (Liu et al., 2013), organizational citizenship behavior (Chen et al., 2013), and employee creativity (Gong et al., 2009). Sample items, rated on a 7-point scale (1 = *strongly disagree*, 7 = *strongly agree*), include “This employee makes an important contribution to the overall performance of our work unit” and “The performance of this employee always meets my requirements/expectations.” The Cronbach's alpha for the scale was 0.85 for Time 1 and 0.93 for Time 2.

#### Control Variables^5^

We controlled for several variables, including two dummies indicating two comparisons between three organizations, supervisors' sex (1 = male, 0 = female), age, and education years, as well as employees' sex (1 = male, 0 = female), age, and education years, because these demographics have been found to influence supervisor-rated employee performance (Djurdjevic and Wheeler, 2014). In addition, we controlled for sex similarity between the supervisors and employees (1 = same sex, 0 = different sex), as well as supervisor perception of similarity with the employees, because demographic and perception of similarity can influence supervisor-rated employee performance (Tepper et al., 2011). To measure the supervisor perception of similarity, we adopted the six-item scale from Liden et al. (1993), rating the items on a 7-point scale (1 = *strongly disagree*, 7 = *strongly agree*). One sample item in this scale is “My employee and I are similar in terms of our outlook, perspective, and values.” Cronbach's alpha was 0.91. We also controlled for the interaction frequency between supervisors and employees to rule out the possibility that familiarity can increase performance evaluation (Reichers, 1987). We used one item from Anderson and West (1998) to measure whether the supervisors frequently interacted with the employees, by asking “How often do you interact with this employee while at work?” (1 = *not at all*, 7 = *very often*). Lastly, we controlled for the employees' original BMI at Time 1 as employees' original body type is likely to affect supervisor-rated employee performance (Levine and Schweitzer, 2015), which also avoided possible problems of using change scores (Cronbach and Furby, 1970; Maxwell and Howard, 1981; Wang, 2007; Bodner and Bliese, 2018; Parke et al., 2020).

## Analytical Strategies

**Figure 1:**
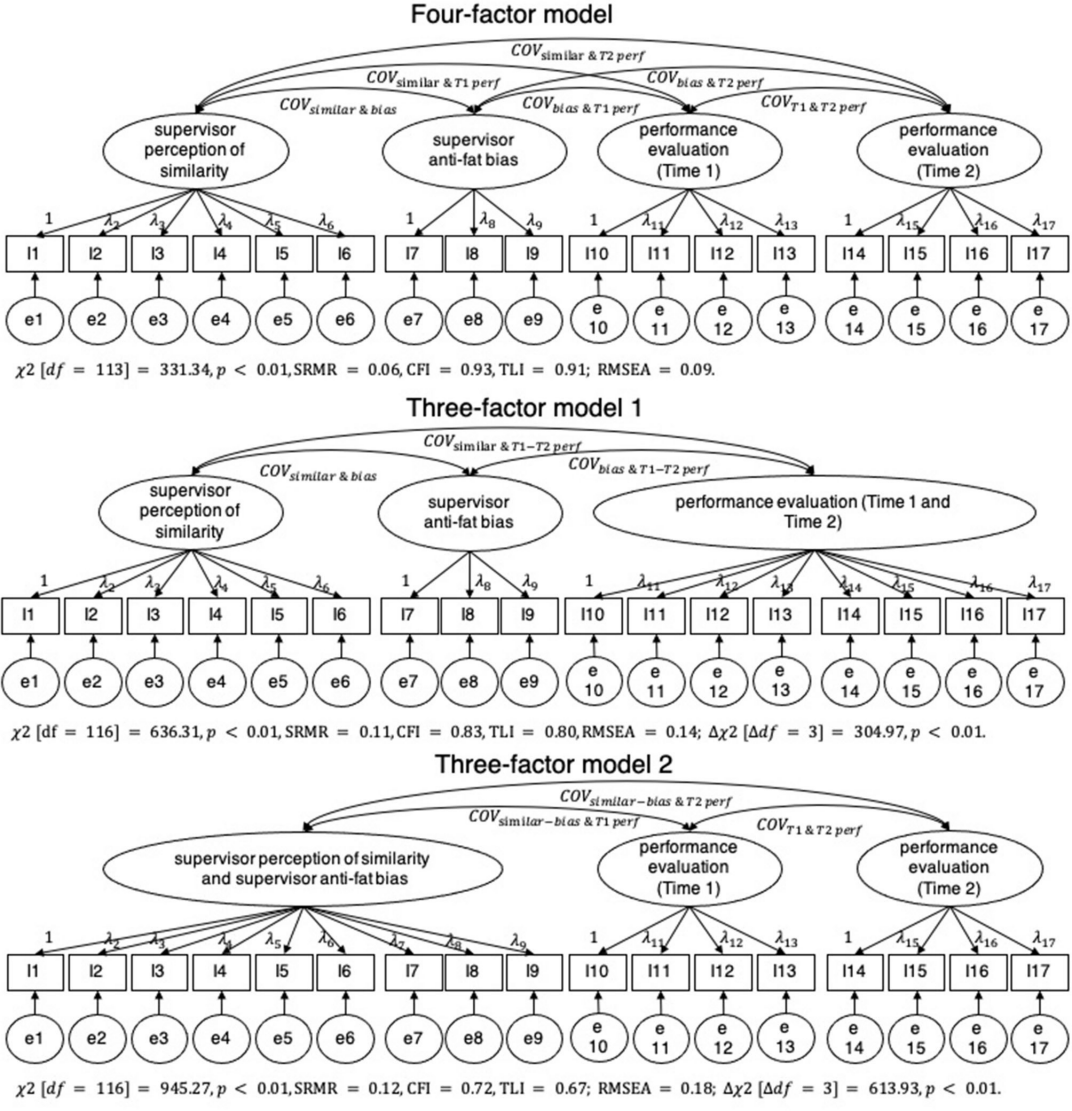
The confirmatory factor analysis. I, item; e, error variance; λ, factor loading; COV, latent factor covariance; similar, supervisor perception of similarity; bias, supervisor anti-fat bias; T, Time; perf, performance evaluation; T1–T2 perf, combination of Time-1 and Time-2 performance evaluation; similar–bias, combination of supervisor perception of similarity and supervisor anti-fat bias; χ^2^, Chi-square Values; df, Degree of Freedom; SRMR, Standardized Root Mean Square Residual; TLI, Tucker–Lewis Index; CFI, Comparative Fit Index; RMSEA, Root Mean Square Error of Approximation.

We first conducted confirmatory factor analyses (CFA) using Mplus 8.0 to validate the distinctiveness of the multi-item variables in our research model. Four latent constructs were involved in the analysis: supervisor perception of similarity, supervisor anti-fat bias, Time-1 performance evaluation, and Time-2 performance evaluation. We derived the conventional chi-square-based fit indexes, including standardized root mean square residual (SRMR), comparative fit index (CFI), Tucker–Lewis index (TLI), and root mean square error of approximation (RMSEA), and illustrated the estimated models in Figure 1.

**Figure 2:**
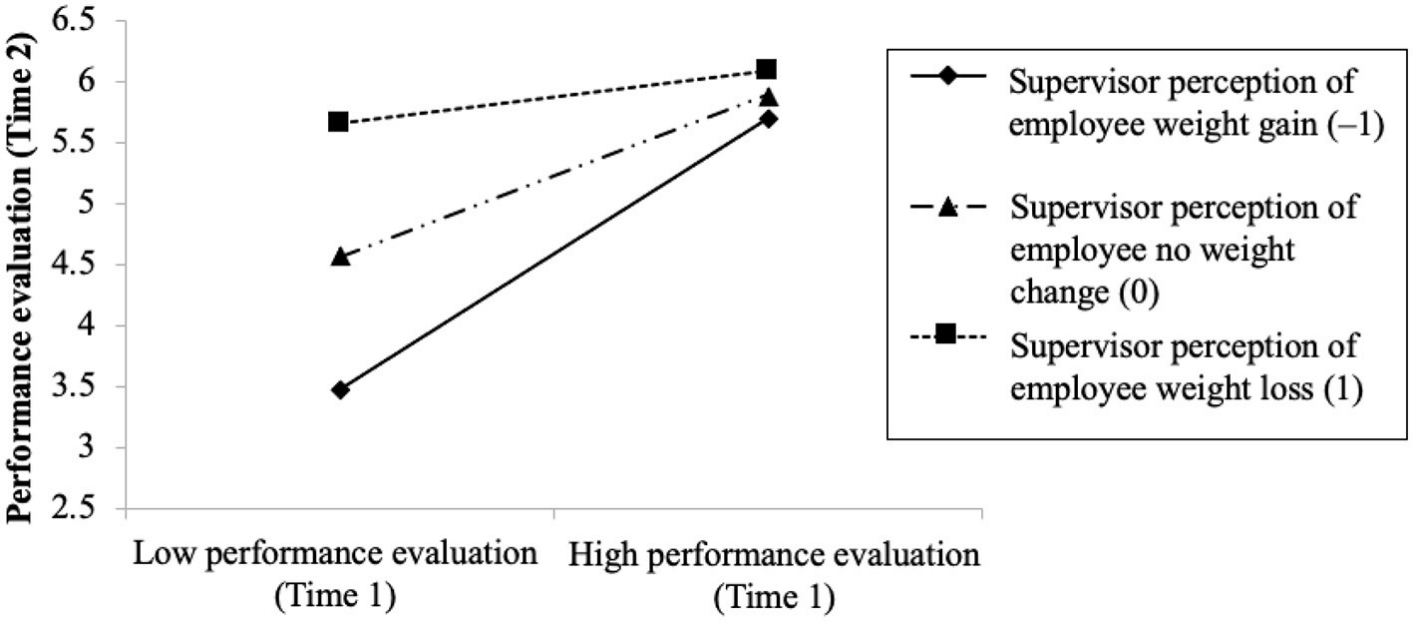
The moderating effect of supervisor perception of employee weight change on the relationship between Time 1 performance evaluation and Time 2 performance evaluation.

We then tested all the hypotheses using SPSS 23.0. First, to examine the hypothesized moderating effect of supervisor perception of weight change (Hypothesis 1), we performed an ordinary least squares (OLS) regression on Time-2 performance evaluation by entering the main effects (Time-1 performance evaluation and supervisor perception of weight change), the two-way interaction, and the control variables, with predictors centered around their respective means (Aguinis, 1995). Following the recommendation of Dawson and Richter (2006), we applied slope difference tests to confirm the slopes of the regression lines are significantly different. Both results were reported in the texts. Consistent with the recommendation of Cohen et al. (2013), we plotted regression lines at three different perceptions of weight change (weight gain = −1, no weight-change = 0, weight loss = 1) in Figure 2 to illustrate the interaction effects.

We subsequently tested whether employee actual weight (BMI) change is significantly related to supervisor perception of weight change (Hypothesis 2) by running an OLS regression on supervisor perception of weight change with predictors including employee actual weight change and control variables. The results were presented in **Table 3**.

**Figure 3:**
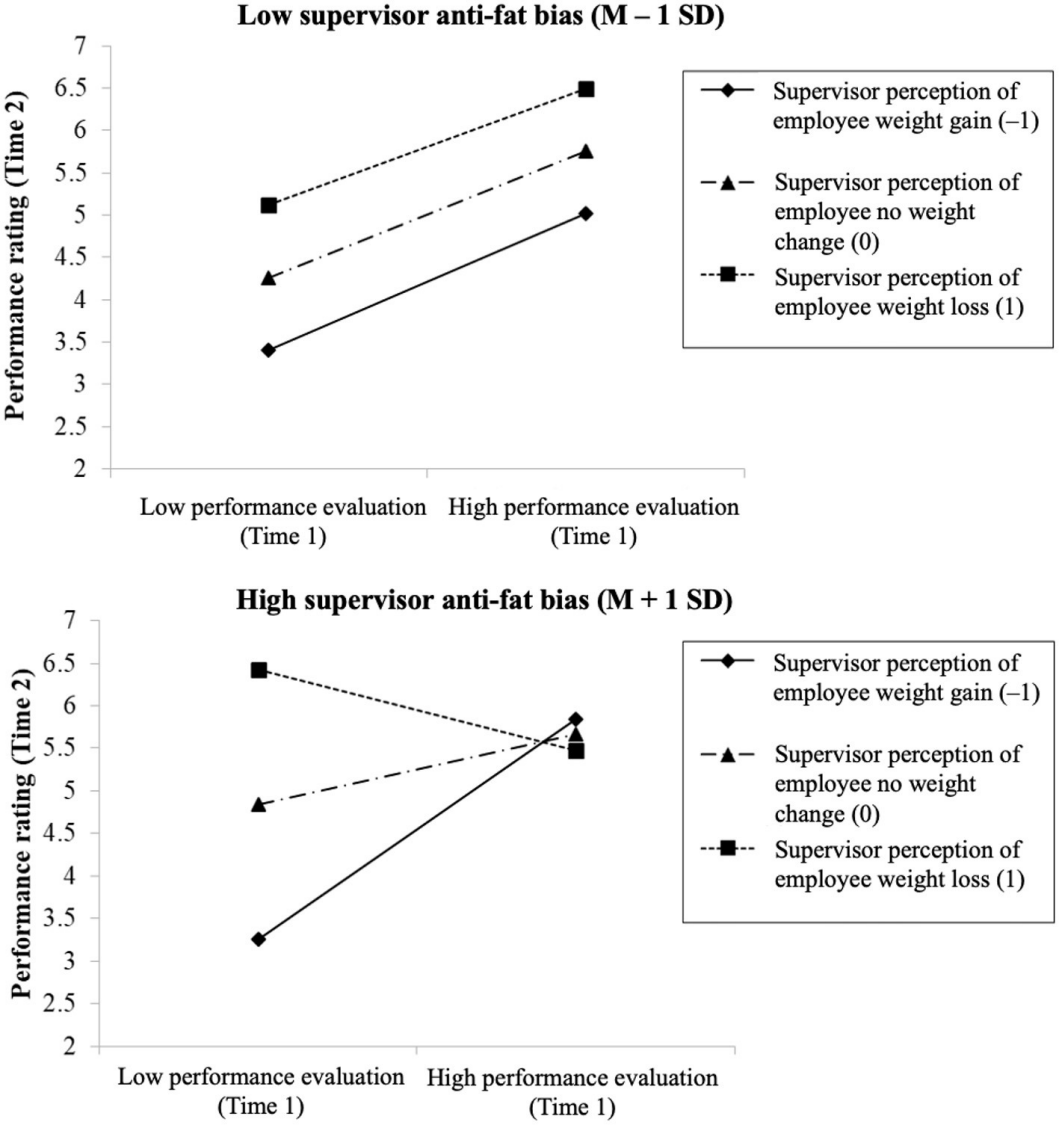
The moderating effect of supervisor anti-fat bias on the interaction between Time 1 performance evaluation and supervisor perception of employee weight change.

Finally, we investigated the hypothesized three-way interacting effects of Time-1 performance evaluation, supervisor perception of weight change, and supervisor anti-fat bias on Time-2 performance evaluation (Hypothesis 3). We ran an OLS regression on Time-2 performance evaluation by entering the main effects (Time-1 performance evaluation, supervisor perception of weight change, and supervisor anti-fat bias), the three two-way interactions, the three-way interaction, and the control variables. Results are shown in **Table 3**. Following the recommendations of Aguinis (1995), predictors were centered before performing regression analyses. Consistent with the recommendation of Cohen et al. (2013), we plotted regression lines at high, mean, and low levels of supervisor anti-fat bias (Mean ± 1 SD) in Figure 3 to facilitate the interpretation of the interaction effects.

## Results

### Confirmatory Factor Analysis

To verify the variables measured in our research captured separate constructs, we conducted confirmatory factor analyses. As shown in Figure 1, the four-factor model (e.g., supervisor perceived similarity, supervisor anti-fat bias, Time-1 performance evaluation, and Time-2 performance evaluation) not only fit the data fairly (χ^2^ [*df* = 113] = 331.34, *p* < 0.01, SRMR = 0.06, CFI = 0.93, TLI = 0.91; RMSEA = 0.09) but was also better than a few alternative three-factor models. Examples of such models had combined Time-1 and Time-2 performance evaluations (χ^2^ [*df* = 116] = 636.31, *p* < 0.01, SRMR = 0.11, CFI = 0.83, TLI = 0.80, RMSEA = 0.14; Δχ^2^ [Δ*df* = 3] = 304.97, *p* < 0.01), and a model with combined supervisor perceived similarity and supervisor anti-fat bias (χ^2^ [*df* = 116] = 945.27, *p* < 0.01, SRMR = 0.12, CFI = 0.72, TLI = 0.67; RMSEA = 0.18; Δχ^2^ [Δ*df* = 3] = 613.93, *p* < 0.01), thereby providing support for the construct validity.

### Hypothesis Testing

The descriptive statistics, reliability coefficients, and correlations among the studied variables are reported in Table 1. The percentages of nominal variables, such as sex, sex similarity, and supervisor perception of employee weight change, are presented in Table 2. We conducted further testing through regression analyses and report the results in Table 2. In general, the *R*^2^ for each regression was calculated. Endogenous variables explained 16% of the variance in supervisor perception of employee weight change and 50% of the variance in Time-2 performance evaluations. These results explain a sizable portion of the variance in our dependent variables.

**Table 1:** Descriptive statistics, reliability coefficients, and correlations.

**Variable**	**M**	**SD**	**1**	**2**	**3**	**4**	**5**	**6**	**7**	**8**	**9**	**10**	**11**	**12**	**13**	**14**	**15**
**Control variables**																	
1. Employee sex (1 = male)	0.81	0.40	—														
2. Employee age	23.99	2.95	−0.09	—													
3. Employee education years	16.23	1.82	−0.03	0.31^**^	—												
4. Supervisor sex (1 = male)	0.88	0.33	0.09	−0.32^**^	0.00	—											
5. Supervisor age	33.73	7.96	−0.08	0.09	0.15*	−0.06	—										
6. Supervisor education years	15.84	1.72	−0.12	0.25^**^	0.36^**^	−0.01	−0.04	—									
7. Gender similarity (1 = same sex)	0.75	0.43	0.65^**^	−0.19^**^	−0.03	0.41^**^	0.04	−0.15*	—								
8. Supervisor perception of similarity	4.61	1.07	−0.16^*^	0.02	0.05	−0.01	−0.05	0.12	−0.06	0.91							
9. Interaction frequency	5.56	1.12	−0.03	0.00	0.09	−0.05	0.05	0.03	−0.05	0.11	—						
10. Employee BMI (Time 1)	23.95	1.01	0.11	0.18^**^	0.15^*^	−0.09	−0.07	0.14^*^	0.00	0.04	0.18^**^	—					
**Studied variables**																	
11. Supervisor anti-fat bias (Time 1)	2.19	1.02	0.13^*^	−0.21^**^	−0.20^**^	0.09	−0.14^*^	−0.18^**^	0.12	−0.04	−0.27^**^	−0.28^**^	0.92				
12. Performance evaluation (Time 1)	5.01	1.02	0.00	−0.24^**^	−0.12	0.13^*^	0.02	−0.19^**^	0.17^*^	0.45^**^	0.09	−0.14^*^	0.10	0.85			
13. Employee BMI change^a^ (Time 2)	1.51	1.10	0.04	0.03	0.04	−0.05	−0.01	−0.02	0.00	−0.10	−0.14^*^	−0.06	0.05	−0.13	—		
14. Supervisor perception of employee weight change^b^ (Time 2)	−0.04	0.51	−0.13	−0.06	−0.10	0.10	0.06	−0.06	−0.07	0.21^**^	0.11	0.03	−0.19^**^	0.11	−0.19^*^	—	
15. Performance evaluation (Time 2)	5.19	1.22	−0.02	−0.19^**^	−0.09	0.17^**^	0.05	−0.09	0.06	0.20^**^	0.09	−0.14^*^	0.03	0.54^**^	−0.17^*^	0.38^**^	0.93

*N = 226;*

a *Time 2 = 6 months later*.

b *Supervisor perception of employee weight change: −1 = weight gain, 0 = no weight change, 1 = weight loss. Cronbach's alphas appear on the diagonal*.

* *p < 0.05*,

** *p < 0.01*.

**Table 2:** Percentages of nominal variables.

	**Number**	**%**
Employee sex		
Employee sex (1 = male)	182	81
Employee sex (0 = female)	44	19
Supervisor sex		
Supervisor sex (1 = male)	198	88
Supervisor sex (0 = female)	28	12
Gender similarity		
Gender similarity (1 = same sex)	170	75
Gender similarity (0 = different sex)	56	25
Supervisor perception of employee weight change		
Weight gain = −1	35	16
No weight change = 0	166	73
Weight loss = 1	25	11

*N = 226. Percentages are based on the total number of dyads (N = 226) and add to 100% for each variable*.

In Hypothesis 1, we argued that supervisor perception of employee weight change should moderate the relationship between Time-1 and Time-2 performance evaluations. Time-2 performance evaluations were regressed in the SPSS using OLS regression method on the controls, as well as employee BMI at Time 1, employee BMI change, supervisor perceptions of employee weight change, Time-1 performance evaluations, and the interaction between supervisor perception of employee weight change and Time 1 performance evaluations. The results suggest the interaction term was significant (γ = −0.44, *p* < 0.01; Δ*R*^2^ for adding the interaction term = 0.04, *p* < 0.01). Such a result indicates the positive relationship between Time-1 and Time-2 performance evaluations would be mitigated if supervisors perceived a weight loss (coded as 1), and the positive relationship would be enhanced if supervisors perceived a weight gain (coded as −1). To facilitate the interpretation of such results, we followed the recommendation of Cohen et al. (2013) to plot regression lines at three different perceptions of weight change (weight gain = −1, no weight-change = 0, weight loss = 1) in Figure 2 to illustrate the interaction effects. Following the recommendation of Dawson and Richter (2006), we applied slope difference tests to confirm the slopes of the regression lines shown in the Figure 2 are significantly different. From the figure, the simple slope for no weight-change perception was significantly positive (γ = 0.65, *p*< *0.0*1). However, the simple slope for weight-loss perception became less sharp (γ = 0.21, *p*< *0.0*5) because the low performance evaluation in Time 1 became more positive in Time 2, whereas high performance evaluation in Time 1 remained relatively similar. Meanwhile, the simple slope for weight-gain perception became considerably sharper (γ = 1.09, *p*< *0.0*1) because the low performance evaluation in Time 1 became even lower, whereas high performance evaluation in Time 1 remained the same. Therefore, Hypothesis 1 was supported, such that the moderating effects varied if the change perception differed among weight gain, no weight change, and weight loss, as well as between high vs. low Time-1 performance evaluation.

In Hypothesis 2, we assumed a significant association between the employee actual BMI change and supervisor perception of employee weight change. The supervisor perception of employee weight change was regressed again in the SPSS using OLS regression method on the same set of controls and on employee BMI change. The results (Model 1 of Table 3) indicate a negative and significant relationship (γ = −0.07, *p*< *0.0*5; Δ*R*^2^ for adding employee BMI change = 0.02, *p* < 0.05) because we coded weight-change perception such that a high value indicates weight loss, whereas a high value of BMI change indicates weight gain. As such, the result supports Hypothesis 2.

**Table 3:** Unstandardized coefficients for the hypothesized model.

**Variables**	**Supervisor perception of employee weight change (Time 2)**	**Performance evaluation (Time 2)**
	**Model 1**	**Model 2**
	**b (SE)**	**b (SE)**
Intercept	0.07 (0.08)	5.15^**^ (0.20)
**Controls**		
Company 2 (vs. Company 1)	−0.14 (0.10)	0.14 (0.21)
Company 3 (vs. Company 1)	−0.14 (0.10)	0.12 (0.21)
Employee sex (1 = male)	−0.01 (0.11)	0.22 (0.22)
Employee age	−0.01 (0.01)	0.00 (0.03)
Employee education years	−0.03 (0.02)	−0.01 (0.04)
Supervisor sex (1 = male)	0.24^*^ (0.11)	0.23 (0.23)
Supervisor age	0.00 (0.00)	0.00 (0.01)
Supervisor education years	−0.02 (0.02)	0.06 (0.04)
Gender similarity (1 = same sex)	−0.08 (0.11)	−0.29 (0.22)
Supervisor perception of similarity	0.09^**^ (0.03)	−0.17^*^ (0.07)
Interaction frequency	0.01 (0.03)	0.07 (0.06)
Employee BMI (Time 1)	−0.00 (0.03)	−0.10 (0.07)
**Main studies variables**		
Supervisor anti-fat bias (SAFB, Time 1)	−0.09^*^ (0.04)	0.11 (0.07)
Employee BMI change (Time 2)	−0.07^*^ (0.03)	−0.02 (0.06)
Performance evaluation (PE, Time 1)		0.59^**^ (0.08)
Supervisor perception of employee weight change (SPEWC, Time 2)		0.76^**^ (0.14)
**Interactions**		
PE × SPEWC		−0.46^**^ (0.12)
PE × SAFB		−0.16^*^ (0.08)
SPEWC × SAFB		−0.08 (0.14)
PE × SPEWC × SAFB		−0.43^**^ (0.14)
*R*^2^	0.16^**^	0.50^**^

*N = 226*.

* *p < 0.05*,

** *p < 0.01*.

We then examined the general role of supervisor anti-fat bias as boundary conditions in the last hypothesis of our study. In Hypothesis 3, we suggested supervisor anti-fat bias should moderate the interaction between supervisor perception of employee weight change and the Time-1 performance evaluation on the Time-2 performance evaluation. Time-2 performance evaluations were regressed in the SPSS using OLS regression method on the variables shown in the third column of Table 3. The results show the three-way interaction term among Time-1 performance evaluations, supervisor perception of employee weight change, and supervisor anti-fat bias was significant (γ = −0.43, *p* < 0.01; Δ*R*^2^ for adding the three-way interaction term = 0.02, *p* < 0.01). Again, to facilitate the interpretation of such results, we followed the recommendation of Cohen et al. (2013) to delineate the interaction in Figure 3 under different values of supervisor anti-fat bias (M± 1SD). Also following the recommendation of Dawson and Richter (2006), we applied slope difference tests to confirm the slopes of the regression lines shown in the Figure 3 are significantly different. From the figure, the interaction effect between Time-1 performance evaluations (X) and supervisor perception of weight change (M_1_) was only significant when the supervisor had a high anti-fat bias (γ for the X^*^M_1_ = −0.89, *p* < 0.01; shown in the lower part of Figure 2). On the contrary, when the supervisor had a low anti-fat bias (shown in the upper part of Figure 2), the interaction term of X^*^M_1_ became insignificant (γ for the X^*^M_1_ = −0.05, *not significant*), thereby indicating there was no weight bias effect under this condition (M−1SD). As such, Hypothesis 3 was supported.

## Discussion

Our study found that if employees' weight changed over 6 months, the Time-1 performance evaluation would be prone to change, such that the association between a new (Time 2) performance evaluation and an evaluation from Time 1 may be altered. The supervisor perception of the employee weight change during that period played an important role. Specifically, supervisor perception of employee weight loss made Time-1 low performance evaluations more positive, whereas weight gain made them more negative. If supervisors provided high Time-1 performance evaluations of employees, the perception of a weight change (weight gain and loss) did not significantly change the high Time-1 performance evaluation at Time 2. Finally, such a moderating effect of supervisor perception of employee weight change was only significant when supervisors had a high level of anti-fat bias.

### Theoretical Implications

This study offers several important theoretical implications for the current literature. First, we developed a systematic view of weight change for weight-bias research by introducing the phase-shifting perspective (Soenen et al., 2017; Stouten et al., 2018). Our research found that weight change transcends original weight in predicting supervisors' most-recent evaluations, indicating that supervisors may be more sensitive to new information, and their previous judgments or evaluations can be altered by more recent perceptions of weight change (Sharif and Oppenheimer, 2016). For example, supervisors may hold more negative attitudes toward weight-gained employees than originally obese employees, as they observed the weight gain process and were more likely to attach negative attributes to weight-gained employees. Thus, we highlight *change* as an important and natural phenomenon of body weight, which can be susceptible to weight bias. However, weight-bias research has thus far largely neglected the changing nature of weight. More importantly, previous research has failed to provide a theoretical underpinning to understand weight change. On the basis of the phase-shifting perspective (Lind, 2001; Proudfoot and Lind, 2015; Audrey Korsgaard et al., 2018), we significantly extend the static “fixed” view of weight bias to a changing one. By doing so, our research is among the first to focus on weight change and examine how it is likewise influenced by weight bias through a systematic and theoretical lens. One of the major benefits of our research is that the consequence of workplace weight bias includes not only supervisors' discrimination against overweight employees, but also their biased reaction to employee weight changes. Future research can consider incorporating the phase-shifting perspective to study weight bias and weight change.

Second, our research highlights two important factors that may explain or influence the process by which weight change actually alters the initial evaluation. The first one is the *perception* of weight change. The phase-shifting perspective, which originated from the heuristic theory (Kahneman and Frederick, 2002; Evans, 2008), emphasizes the role of the *perception of a change* in triggering analytic information-processing that can potentially alter one's previous evaluation (Cropanzano and Rupp, 2003; Skarlicki and Rupp, 2010). Our research confirms the importance of such perception in performance-evaluation processes. Although such perception can only occur after an actual weight change, it seems more proximal to evaluation alteration than the actual change. Furthermore, the effects of weight-gain and weight-loss perceptions can be dramatically different. Compared with the perception of no weight change, weight-gain and weight-loss perceptions significantly altered the prior-change (Time 1) performance evaluation, although the directions differed. The low performance evaluation at Time 1 became higher with weight-loss perception but lower with weight-gain perception. As such, our research confirms the potential benefiting role of losing weight as a coping strategy to escape the weight stigma (Puhl et al., 2005; Levine and Schweitzer, 2015). Our research also warns weight-bias researchers that being overweight and gaining more weight may trigger others' weight bias.

Such findings not only reveal the subtlety and complexity of human heuristics, but also advance the phase-shifting perspective, given that previous literature tends to treat the phase-shifting perception simply as one dichotomy, that is, perceiving a change or no change (Soenen et al., 2017). Instead, our study advances the phase-shifting perspective by providing it with a more sophisticated view. Our study indicates *change* should be placed into context to understand its effects. For instance, weight change should include weight loss and weight gain. Such a sophisticated view of change reveals new findings that were missing from the previous dichotomous approach; that is, weight-loss perception brings different consequences than weight-gain perception, although both are change perceptions. As such, future research that adopts the phase-shifting perspective may likewise consider examining the content of the change perception rather than treating it solely as the overall perception of whether a change occurs.

The second factor important to weight-change research is the observers' anti-fat bias that, in our study, significantly moderates the effect of the perception of weight change on the alteration in performance evaluation. People can perceive a change and process its content, although the change may be irrelevant to the evaluation process. If the observer thinks the change is irrelevant to the context, then his following evaluation may not be influenced. Our research shows that only those who possess an anti-fat bias will believe overweight persons are too obese to perform well in their jobs and thus react negatively to others' weight change by altering their prior-change performance evaluation. Such findings not only provide a boundary condition for weight change to influence evaluation, but also further the current weight-bias literature by confirming the important boundary role of anti-fat bias. Future studies can consider exploring the antecedents of anti-fat bias or interventions that can stop such discrimination.

Third, our study contributes to the performance-evaluation literature. Previous literature tends to treat performance evaluation as a static, cross-sectional, and one-time judgment, thereby largely neglecting its changing nature (Becker and Cropanzano, 2011). Peterson et al. (2011) found that performance evaluation changes over time and can thus serve as an important source of new performance evaluation. In view of this argument, our study advances the changing views of performance evaluation (e.g., Ferris et al., 2008) by examining how evaluation-condition changes can lead to evaluation changes. As previously stated, the phase-shifting perspective can help in understanding the longitudinal change of one's performance evaluation. Our study suggests weight change can contribute to a perception of relevant condition change. Specifically, if the observer possesses anti-fat bias and if weight can shape the prior-change performance evaluation, the observee's weight change can likewise lead to an alteration in the observer's evaluations. Weight-change perception may also interact with prior-change performance evaluation to shape the new performance evaluation. As such, our study sheds light on how a change in the performance-evaluation context can be based on prior-change performance evaluation to form a new evaluation. Our study provides a new avenue for future performance researchers to consider the dynamic change in shaping new evaluations.

### Practical Implications

Our research provides important practical implications for organizational practitioners and employees. First, as our study found that supervisor-rated performance could be contaminated by weight bias, it is crucial for organizations to design leadership training programs to improve performance appraisal accuracy carefully. We suggest training supervisors with objective, behavior-based rating instruments, which can enhance rating accuracy (Pulakos, 1984). For example, Borman (1979) proposed a training approach called frame-of-reference (FOR) training, which involves emphasizing performance dimensions, providing samples of behavioral incidents representing each dimension, indicating the level of performance defined by each incident, and supplying feedback by using these standards to evaluate performance (Woehr, 1994). Overall, organizations could provide supervisors with this training to improve their rating accuracy and avoid possible bias.

Similarly, in conjunction with other workplace anti-discrimination training, it is important for organizations to train leaders and subordinates alike to both recognize and mitigate anti-fat biases at work (Ruggs et al., 2015). Anti-fat bias can be both known and unconscious, therefore training all employees to recognize their implicit and explicit biases can help mitigate discrimination from the top-down and bottom-up. Organizations requiring anti-fat bias training also send a strong message to their employees, shareholders, and customers that such discrimination will not be tolerated, which is another major step in bringing awareness to this rampant yet neglected issue, as well as decreasing instances of anti-fat workplace prejudice.

Our findings also emphasize the need for legislation to address the pervasiveness of anti-fat discrimination. In the United States, only Michigan has passed legislation explicitly addressing weight discrimination in the workplace. Even so, Kirkland (2006) found instances where this legislation actually upheld discrimination rather than prohibited it. As our study adds to the literature highlighting the rampancy of anti-fat bias and its detrimental effects, state and national governments should work to pass an effective anti-fat discrimination policy. Just as the Civil Rights Act (1964) prohibits workplaces in the United States from discriminating on the grounds of ethnicity, race, color, religion, sex, national origin, disability, or age, governmental policy should also protect against weight discrimination. Such legislative enactment can also help to expedite organizational action and training to prevent anti-fat discrimination.

### Future Directions and Limitations

Our study has several limitations that could help shed light on future research directions. First, the current study focused on subjective performance evaluation instead of objective performance because, in today's workplace, few jobs are designed in a way that facilitates objective performance measurement (Tangen, 2003). Results showed that supervisors with anti-fat bias are more likely to give employees who gain weight lower performance evaluations. We interpreted the lower performance evaluations as the outcome of the interaction of supervisor weight bias, employee weight change, and previous performance evaluation. However, there could be an alternative explanation: the employee who gains weight performs objectively worse than those who do not gain weight. Without an objective measure of employee performance, it is hard to determine whether the supervisor-rated employee performance is influenced by supervisor weight bias or/and by their objective performance. Therefore, our research was only exploratory and indicated a primary relationship between weight change and subjective performance evaluation. Objective measures of employee performance are needed in follow-up studies to address further the underlying logic of the relationship between weight change and performance.

Another possible limitation is the demand characteristic in the measurement rated by supervisors. We asked supervisors to provide ratings on employee performance and their own anti-fat bias in the same wave of survey (Time 1), which might raise the concern of demand characteristic. According to Orne's (2002) theory, participants are trying to meet the research demands as well as social expectations, which is defined as *demand characteristic*. Demand characteristic will result in the socially desirable response bias (i.e., responding positively to shape a positive impression of them; Nichols and Maner, 2008). In our study, the demand characteristic might lead supervisors to report lesser weight bias and hide the relationship between an employee weight change and their performance evaluation, which disconfirms the hypotheses of our study. As such, the demand characteristic in supervisor-report measurement should have reduced the statistical power of our measurement. Although the current study still found significant effects of supervisor weight bias moderating the relationship between employee weight change and performance evaluation, future research on weight bias could try to avoid such a problem to get a more accurate estimation of the effect sizes of weight bias (e.g., measuring and controlling social desirability; Wang et al., 2015).

Finally, future research could dig into the effects of weight change on performance evaluation from alternative perspectives like the anchoring and adjustment heuristic. According to Tversky and Kahneman (1974), one's estimates are biased toward different initial points, which they defined as *anchors*. As such, people tend to adjust their final judgment toward the starting estimates. According to the perspective of our study, an employee who changed their BMI from overweight to normal would have higher performance evaluations than someone who didn't change BMI. However, from the theoretical perspective of the anchoring and adjustment heuristic, an employee who changed their BMI from overweight to normal would receive lower performance evaluations from a supervisor with anti-fat bias than someone who didn't change BMI, because the original judgment was “sticky.” Therefore, it would be interesting for future studies to test the anchoring and adjustment heuristic vs. weight bias effects in performance evaluation.

## Data Availability Statement

The original contributions presented in the study are included in the article/supplementary material, further inquiries can be directed to the corresponding author.

## Ethics Statement

Ethical review and approval was not required for the study on human participants in accordance with the local legislation and institutional requirements. Written informed consent for participation was not required for this study in accordance with the national legislation and the institutional requirements.

## Author Contributions

YJ, QH, and HL contributed to research idea, theoretical construction, designed the experiment, and collected data. CP contributed to the interpretation of data and writing and revising of the work.

## Conflict of Interest

The authors declare that the research was conducted in the absence of any commercial or financial relationships that could be construed as a potential conflict of interest.

## Footnotes

^1^Michigan's Elliott Larsen Act: 37.2102 Recognition and declaration of civil right. Sec. 102. (1) The opportunity to obtain employment, housing, and other real estate, and the full and equal utilization of public accommodations, public service, and educational facilities without discrimination because of religion, race, color, national origin, age, sex, height, weight, familial status, or marital status as prohibited by this act, is recognized and declared to be a civil right.

^2^Since self-report height and weight were adopted to cultivate BMI, there could be a self-report bias (Elgar and Stewart, 2008). The self-report BMI is 1.16 smaller than the measured BMI based on a large study conducted by Elgar and Stewart (2008).

^3^Using change scores that are generated by subtracting baseline scores from final scores can lead to incorrect conclusions because they are likely to miss out the main effects of the variables (Cronbach and Furby, 1970). Therefore, we followed the instructions and analysis procedures of previous research (Cronbach and Furby, 1970; Maxwell and Howard, 1981; Wang, 2007; Bodner and Bliese, 2018; Parke et al., 2020) to control the Time-1 employee BMI when testing the effects of employee weight change.

^4^To give a more general picture of the relationship between supervisor-rated employee performance and employee objective performance in different studies using different scales, the meta-analysis showed that the overall mean corrected correlation between the two can be 0.45 (Rich et al., 1999).

^5^The pattern of results stayed identical after omitting the controls.

## References

[B1] Agerström J. Rooth D. O. (2011). The role of automatic obesity stereotypes in real hiring discrimination. J. Appl. Psychol. 96, 790–805. 10.1037/a0021594 21280934

[B2] Aguinis H. (1995). Statistical power with moderated multiple regression in management research. J. Manag. 21, 1141–1158. 10.1177/014920639502100607

[B3] Anderson N. R. West M. A. (1998). Measuring climate for work group innovation: development and validation of the team climate inventory. J. Organ. Behav. 19, 235–258.

[B4] Audrey Korsgaard M. Kautz J. Bliese P. Samson K. Kostyszyn P. (2018). Conceptualizing time as a level of analysis: new directions in the analysis of trust dynamics. J. Trust Res. 8, 142–165. 10.1080/21515581.2018.1516557

[B5] Becker W. J. Cropanzano R. (2011). Dynamic aspects of voluntary turnover: an integrated approach to curvilinearity in the performance–turnover relationship. J. Appl. Psychol. 96, 233–246. 10.1037/a0021223 20853945

[B6] Bento R. F. White L. F. Zacur S. R. (2012). The stigma of obesity and discrimination in performance appraisal: a theoretical model. Int. J. Hum. Resour. Manag. 23, 3196–3224. 10.1080/09585192.2011.637073

[B7] Bernardin H. J. Thomason S. Buckley M. R. Kane J. S. (2016). Rater rating-level bias and accuracy in performance appraisals: the impact of rater personality, performance management competence, and rater accountability. Hum. Res. Manag. 55, 321–340. 10.1002/hrm.21678

[B8] Bhutani S. Kahn E. Tasali E. Schoeller D. A. (2017). Composition of two-week change in body weight under unrestricted free-living conditions. Physiol. Rep. 5:e13336. 10.14814/phy2.13336 28676555 PMC5506524

[B9] Biernacki P. (1986). Pathways From Heroin Addiction: Recovery Without Treatment. Philadelphia, PA: Temple University Press.

[B10] Blaine B. E. DiBlasi D. M. Connor J. M. (2002). The effect of weight loss on perceptions of weight controllability: implications for prejudice against overweight people. J. Appl. Biobehav. Res. 7, 44–56. 10.1111/j.1751-9861.2002.tb00075.x

[B11] Bodner T. E. Bliese P. D. (2018). Detecting and differentiating the direction of change and intervention effects in randomized trials. J. Appl. Psychol. 103, 37–53. 10.1037/apl0000251 28805426

[B12] Borman W. C. (1979). Format and training effects on ratings accuracy and rater errors. J. Appl. Psyhcol. 64, 410–412. 10.1037/0021-9010.64.4.410

[B13] Brockner J. Ackerman G. Greenberg J. Gelfand M. J. Francesco A. M. Zhen X. C.. (2001). Culture and procedural justice: the influence of power distance on reactions to voice. J. Exp. Soc. Psychol. 37, 300–315. 10.1006/jesp.2000.1451

[B14] Brownell K. D. Puhl R. M. Schwarz M. B. Rudd L. (2005). Weight Bias: Nature, Consequences, and Remedies. New York, NY: Guilford Press.

[B15] Bulik C. M. Wade T. D. Heath A. C. Martin N. G. Stunkard A. J. Eaves L. J. (2001). Relating body mass index to figural stimuli: population-based normative data for caucasians. Int. J. Obes. 25, 1517–1524. 10.1038/sj.ijo.0801742 11673775

[B16] Carter M. Z. Mossholder K. W. (2015). Are we on the same page? the performance effects of congruence between supervisor and group trust. J. Appl. Psychol. 100, 1349–1363. 10.1037/a0038798 25688640

[B17] Center for Disease Control Prevention (2019). Nutrition, Physical Activity, and Obesity: Data, Trends, and Maps. Available online at: http://www.cdc.gov/obesity/data/adult.html (accessed June 1, 2021).

[B18] Charlesworth T. E. Banaji M. (2019). Patterns of implicit and explicit attitudes: I. Long-term change and stability from 2006 to 2016. Psychol. Sci. 30 174–192. 10.1177/0956797618813087 30605364

[B19] Chen W. A. N. G. Xu-Hong H. Zhang M. L. Yu-Qian B. Yu-Hua Z. Zhong W. H.. (2010). Comparison of body mass index with body fat percentage in the evaluation of obesity in Chinese. Biomed. Environ. Sci. 23, 173–179. 10.1016/S0895-3988(10)60049-9 20708495

[B20] Chen Z. Takeuchi R. Shum C. (2013). A social information processing perspective of coworker influence on a focal employee. Organ. Sci. 24, 1618–1639. 10.1287/orsc.2013.0820

[B21] Chen Z. X. Aryee S. (2007). Delegation and employee work outcomes: an examination of the cultural context of mediating processes in China. Acad. Manage. J. 50, 226–238. 10.5465/amj.2007.24162389

[B22] Cohen J. Cohen P. West S. G. Aiken L. S. (2013). Applied Multiple Regression/Correlation Analysis for the Behavioral Sciences. Mahwah, NJ: Routledge.

[B23] Colella A. Hebl M. King E. (2017). One hundred years of discrimination research in the Journal of Applied Psychology: a sobering synopsis. J. Appl. Psychol. 102, 500–513. 10.1037/apl0000084 28125266

[B24] Cronbach L. J. Furby L. (1970). How should we measure “change”—or should we? Psychol. Bull. 74, 66–80. 10.1037/h0029382

[B25] Cropanzano R. Rupp D. E. (2003). An overview of organizational justice: implications for work motivation, in Motivation and Work Behavior, Porter L. W. Bigley G. A. Steers R. M.. (Burr Ridge, IL: Irwin/McGraw-Hill), 82–95.

[B26] Dawson J. F. Richter A. W. (2006). Probing three-way interactions in moderated multiple regression: development and application of a slope difference test. J. Appl. Psychol. 91, 917–926. 10.1037/0021-9010.91.4.917 16834514

[B27] Devine P. G. (1989). Stereotypes and prejudice: their automatic and controlled components. J. Pers. Soc. Psychol. 56, 5–18. 10.1037/0022-3514.56.1.5

[B28] Dhanani L. Y. Beus J. M. Joseph D. L. (2018). Workplace discrimination: a meta-analytic extension, critique, and future research agenda. Pers. Psychol. 71, 147–179. 10.1111/peps.12254

[B29] Djurdjevic E. Wheeler A. R. (2014). A dynamic multilevel model of performance rating, in Research in Personnel and Human Resources Management, Buckley M. R. Halbesleben J. R. B. Wheeler A. R.. (Bingley: Emerald Group Publishing Limited), 147–176.

[B30] Dovidio J. F. (2010). The SAGE Handjournal of Prejudice, Stereotyping, and Discrimination. Thousand Oaks, CA: Sage Publications.

[B31] Dye G. (2008, April 2). Weight-ism” more widespread than racism. ABC News. Available online at: https://abcnews.go.com/Technology/BeautySecrets/story?id=4568813andpage=1 (accessed April 20, 2020).

[B32] Elgar F. J. Stewart J. M. (2008). Validity of self-report screening for overweight and obesity: evidence from the Canadian community health survey. Can. J. Public Health 99, 423–427. 10.1007/BF03405254 19009930 PMC6975656

[B33] Evans J. S. B. (2008). Dual-processing accounts of reasoning, judgment, and social cognition. Annu. Rev. Psychol. 59, 255–278. 10.1146/annurev.psych.59.103006.093629 18154502

[B34] Fardouly J. Vartanian L. R. (2012). Changes in weight bias following weight loss: the impact of weight-loss method. Int. J. Obes. 36, 314–319. 10.1038/ijo.2011.26 21364528

[B35] Farh J.-L. Cheng B.-S. (1997). Modesty bias in self-rating in Taiwan: impact of item wording, modesty value, and self-esteem. Chin. J. Psychol. 39, 103–118.

[B36] Ferris G. R. Munyon T. P. Basik K. Buckley M. R. (2008). The performance evaluation context: social, emotional, cognitive, political, and relationship components. Hum. Resour. Manage. Rev. 18, 146–163. 10.1016/j.hrmr.2008.07.006

[B37] Fontana F. Furtado Jr O. Mazzardo Jr O. Hong D. de Campos W. (2017). Anti-fat bias by professors teaching physical education majors. Eur. Physchol. Ed. Rev. 23, 127–138. 10.1177/1356336X16643304

[B38] Fryer B. Kirby J. (2005). Fat Chance. Harvard Business Review. Available online at: https://hbr.org/2005/05/fat-chance (accessed April 20, 2020)

[B39] Gaertner S. L. Dovidio J. F. (1986). The aversive form of racism, in Prejudice, Discrimination, and Racism, Dovidio J. F. Gaertner S. L.. (Cambridge, MA: Academic Press), 61–89.

[B40] Gaertner S. L. Dovidio J. F. (2000). The aversive form of racism, in Key Readings in Social Psychology. Stereotypes and Prejudice: Essential Readings, Stangor C.. (New York, NY: Psychology Press), 289–304.

[B41] Gawronski B. Bodenhausen G. V. (2006). Associative and propositional processes in evaluation: an integrative review of implicit and explicit attitude change. Psychol. Bull. 132, 692–731. 10.1037/0033-2909.132.5.692 16910748

[B42] Gong Y. Huang J.-C. Farh J.-L. (2009). Employee learning orientation, transformational leadership, and employee creativity: the mediating role of employee creative self-efficacy. Acad. Manag. J. 52, 765–778. 10.5465/amj.2009.43670890

[B43] Granberg E. M. (2011). Now my ‘old self' is thin” stigma exits after weight loss. Soc. Psychol. Q. 74, 29–52. 10.1177/0190272511398020

[B44] Hart W. Albarracín D. Eagly A. H. Brechan I. Lindberg M. J. Merrill L. (2009). Feeling validated versus being correct: a meta-analysis of selective exposure to information. Psychol. Bull. 135:555. 10.1037/a0015701 19586162 PMC4797953

[B45] Heilman M. E. Block C. J. Stathatos P. (1997). The affirmative action stigma of incompetence: effects of performance information ambiguity. Acad. Manag. J. 40, 603–625. 10.5465/257055

[B46] Howard J. (2008). Negotiating an exit: existential, interactional, and cultural obstacles to disorder disidentification. Soc. Psychol. Q. 71, 177–192. 10.1177/019027250807100206

[B47] Jackson S. E. Beeken R. J. Wardle J. (2014). Perceived weight discrimination and changes in weight, waist circumference, and weight status. Obesity 22, 2485–2488. 10.1002/oby.20891 25212272 PMC4236245

[B48] Jones D. A. Skarlicki D. P. (2013). How perceptions of fairness can change: a dynamic model of organizational justice. Organ. Psychol. Rev. 3, 138–160. 10.1177/2041386612461665

[B49] Judge T. A. Cable D. M. (2011). When it comes to pay, do the thin win? the effect of weight on pay for men and women. J. Appl. Psychol. 96, 95–112. 10.1037/a0020860 20853946

[B50] Kahneman D. Frederick S. (2002). Representativeness revisited: attribute substitution in intuitive judgment, in Heuristic and Biases: The Psychology of Intuitive Judgment, Gilovich T. Griffin D. Kahneman D.. (New York, NY: Cambridge University Press), 49–81.

[B51] King E. B. Hebl M. R. Heatherton T. F. (2005). Theories of stigma: limitations and needed directions, in Weight Bias: Nature, Consequences, and Remedies, Brownell K. D. Puhl R. M. Schwartz M. B. Rudd L.. (New York, NY: The Guilford Press), 109–120.

[B52] Kirkland A. (2006). What's at stake in fatness as a disability? Disabil. Stud. Quart. 26, 1–30. 10.18061/dsq.v26i1.648

[B53] Klesges R. C. Klem M. L. Hanson C. L. Eck L. H. Ernst J. O'Laughlin D.. (1990). The effects of applicant's health status and qualifications on simulated hiring decisions. Int. J. Obes. 14, 527–535. 2401589

[B54] Kolata G. (2016). The shame of fat shaming. New York Times. Available online at: https://www.nytimes.com/2016/10/02/sunday-review/the-shame-of-fat-shaming.html (accessed October 1, 2020).

[B55] Larkin J. C. Pines H. A. (1979). No fat persons need apply: experimental studies of the overweight stereotype and hiring preference. Sociol. Work Occup. 6, 312–327. 10.1177/073088847900600303

[B56] Law K. S. Wong C. Song L. J. (2004). The construct and criterion validity of emotional intelligence and its potential utility in management research. J. Appl. Psychol. 87, 483–496. 10.1037/0021-9010.89.3.483 15161407

[B57] Levine E. E. Schweitzer M. E. (2015). The affective and interpersonal consequences of obesity. Organ. Behav. Hum. Decis. Process. 127, 66–84. 10.1016/j.obhdp.2015.01.002

[B58] Liden R. C. Wayne S. J. Stilwell D. (1993). A longitudinal study on the early development of leader-member exchanges. J. Appl. Psychol. 78, 662–674. 10.1037/0021-9010.78.4.662

[B59] Lind E. A. (2001). Fairness heuristic theory: justice judgments as pivotal cognitions in organizational relations, in Advances in Organizational Justice, Greenberg J. Cropanzano R. S.. (Stanford, CA: Stanford University Press), 56–88.

[B60] Liu J. Hui C. Lee C. Chen Z. X. (2013). Why do I feel valued and why do I contribute? a relational approach to employee's organization-based self-esteem and job performance. J. Manage. Stud. 50, 1018–1040. 10.1111/joms.12037

[B61] Madey S. F. Ondrus S. A. (1999). Illusory correlations in perceptions of obese and hypertensive patients' noncooperative behaviors. J. Appl. Soc. Psychol. 29, 1200–1217. 10.1111/j.1559-1816.1999.tb02036.x

[B62] Maxwell S. E. Howard G. S. (1981). Change scores—necessarily anathema? Educ. Psychol. Meas. 41, 747–756. 10.1177/001316448104100313

[B63] McKee K. Smouse A. D. (1983). Clients' perceptions of counselor expertness, attractiveness, and trustworthiness: initial impact of counselor status and weight. J. Couns. Psychol. 30, 332–338. 10.1037/0022-0167.30.3.332

[B64] Merritt S. Gardner C. Huber K. Wexler B. Banister C. Staley A. (2018). Imagine Me and You, I Do: effects of imagined intergroup contact on anti-fat bias in the context of job interviews. J. Appl. Soc. Psychol. 48, 80–89. 10.1111/jasp.12492

[B65] Moers F. (2005). Discretion and bias in performance evaluation: the impact of diversity and subjectivity. Account. Organ. Soc. 30, 67–80. 10.1016/j.aos.2003.11.001

[B66] Newheiser A. K. Dovidio J. F. (2012). Individual differences and intergroup bias: divergent dynamics associated with prejudice and stereotyping. Pers. Individ. Dif. 53, 70–74. 10.1016/j.paid.2012.02.024

[B67] Nichols A. L. Maner J. K. (2008). The good-subject effect: investigating participant demand characteristics. J. Gen. Psychol. 135, 151–166. 10.3200/GENP.135.2.151-166 18507315

[B68] Nieminen L. R. G. Rudolph C. W. Baltes B. B. Casper C. M. Wynne K. T. Kirby L. C. (2013). The combined effect of ratee's bodyweight and past performance information on performance judgments. J. Appl. Soc. Psychol. 43, 527–543. 10.1111/j.1559-1816.2013.01033.x

[B69] Orne M. T. (2002). On the social psychology of the psychological experiment: with particular reference to demand characteristics and their implications. Prev. Treat. 5, 1522–3736. 10.1037/1522-3736.5.0035a

[B70] Parke M. R. Tangirala S. Hussain I. (2020). Creating organizational citizens: how and when supervisor-versus peer-led role interventions change organizational citizenship behavior. J. Appl. Psychol. 10.1037/apl0000848 33090860

[B71] Pashler H. (1988). Familiarity and visual change detection. Percept. Psychophys. 44, 369–378. 10.3758/BF03210419 3226885

[B72] Peterson S. J. Luthans F. Avolio B. J. Walumbwa F. O. Zhang Z. (2011). Psychological capital and employee performance: a latent growth modeling approach. Pers. Psychol. 64, 427–450. 10.1111/j.1744-6570.2011.01215.x19594228

[B73] Proudfoot D. Lind E. A. (2015). Fairness heuristic theory, the uncertainty management model, and fairness at work, in The Oxford Handjournal of Justice in the Workplace, Cropanzano R. S. Ambrose M. L.. (Oxford: Oxford University Press), 371–385.

[B74] Puhl R. M. Brownell K. Schwartz M. Rudd L. (2005). Coping with Weight Stigma, in Weight Bias: Nature, Consequences, and Remedies, Brownell K.D.. (New York, NY: Guilford Press), 275–284.

[B75] Pulakos E. D. (1984). A comparison of rater training programs: error training and accuracy training. J. Appl. Psychol. 69, 581–588. 10.1037/0021-9010.69.4.581

[B76] Rattan A. Dweck C. S. (2018). What happens after prejudice is confronted in the workplace? how mindsets affect minorities' and women's outlook on future social relations. J. Appl. Psychol. 103, 676–687. 10.1037/apl0000287 29517252

[B77] Reichers A. E. (1987). An interactionist perspective on newcomer socialization rates. Acad. Manage. Rev. 12, 278–287. 10.5465/amr.1987.4307838

[B78] Renehan A. G. Tyson M. Egger M. Heller R. F. Zwahlen M. (2008). Body-mass index and incidence of cancer: a systematic review and meta-analysis of prospective observational studies. Lancet 371, 569–578. 10.1016/S0140-6736(08)60269-X 18280327

[B79] Rich G. A. Bommer W. H. MacKenzie S. B. Podsakoff P. Johnson J. L. (1999). Apples and apples or apples and oranges? a meta-analysis of objective and subjective measures of salesperson performance. J. Pers. Sell. Sales Manag. 19, 41–52.

[B80] Roehling M. V. Pichler S. Bruce T. A. (2013). Moderators of the effect of weight on job-related outcomes: a meta-analysis of experimental studies. J. Appl. Soc. Psychol. 43, 237–252. 10.1111/j.1559-1816.2012.00993.x

[B81] Rudolph C. W. Baltes B. B. Zhdanova L. S. Clark M. A. Bal A. C. (2012). Testing the structured free recall intervention for reducing the impact of bodyweight-based stereotypes on performance ratings in immediate and delayed contexts. J. Bus. Psychol. 27, 205–222. 10.1007/s10869-011-9240-7

[B82] Rudolph C. W. Wells C. L. Weller M. D. Baltes B. B. (2009). A meta-analysis of empirical studies of weight-based bias in the workplace. J. Vocat. Behav. 74, 1–10. 10.1016/j.jvb.2008.09.008

[B83] Ruggs E. N. Hebl M. R. Williams A. (2015). Weight isn't selling: the insidious effects of weight stigmatization in retail settings. J. Appl. Psychol. 100, 1483–1496. 10.1037/apl0000017 25751751

[B84] Salvemini N. J. Reilly R. R. Smither J. W. (1993). The influence of rater motivation on assimilation effects and accuracy in performance ratings. Organ. Behav. Hum. Decis. Process. 55, 41–60. 10.1006/obhd.1993.1023

[B85] Sansone C. Harackiewicz J. M. (2000). Intrinsic and Extrinsic Motivation: The Search for Optimal Motivation and Performance. San Diego, CA: Academic Press.

[B86] Sassi F. (2010). Obesity and the Economics of Prevention: Fit Not Fat. (1. Aufl. ed.). Paris: OECD.

[B87] Schaubroeck J. M. Shen Y. M. Chong S. (2017). A dual-stage moderated mediation model linking authoritarian leadership to follower outcomes. J. Appl. Psychol. 102, 203–214. 10.1037/apl0000165 27786498

[B88] Schwartz M. B. Vartanian L. R. Nosek B. A. Brownell K. D. (2006). The influence of one's own body weight on implicit and explicit anti-fat bias. Obesity 14, 440–447. 10.1038/oby.2006.58 16648615

[B89] Sharif M. A. Oppenheimer D. M. (2016). The effect of relative encoding on memory-based judgments. Psychol. Sci. 27, 1136–1145. 10.1177/0956797616651973 27356963

[B90] Silverstein B. Perdue L. Peterson B. Kelly E. (1986). The role of the mass media in promoting a thin standard of bodily attractiveness for women. Sex Roles 14, 519–532. 10.1007/BF00287452

[B91] Simons D. J. Rensink R. A. (2005). Change blindness: past, present, and future. Trends Cogn. Sci. 9, 16–23. 10.1016/j.tics.2004.11.006 15639436

[B92] Skarlicki D. P. Rupp D. E. (2010). Dual processing and organizational justice: the role of rational versus experiential processing in third-party reactions to workplace mistreatment. J. Appl. Psychol. 95, 944–952. 10.1037/a0020468 20836589

[B93] Soenen G. Melkonian T. Ambrose M. L. (2017). To shift or not to shift? determinants and consequences of phase shifting on justice judgments. Acad. Manag. J. 60, 798–817. 10.5465/amj.2014.0181

[B94] Steiner D. D. Rain J. S. (1989). Immediate and delayed primacy and recency effects in performance evaluation. J. Appl. Psychol. 74, 136–142. 10.1037/0021-9010.74.1.136

[B95] Stolz J. A. Jolicoeur P. (2004). Changing features do not guide attention in change detection: evidence from a spatial cuing paradigm. Psychon. Bull. Rev. 11, 870–875. 10.3758/BF03196714 15732696

[B96] Stouten J. Rousseau D. M. De Cremer D. (2018). Successful organizational change: integrating the management practice and scholarly literatures. Acad. Manag. Ann. 12, 752–788. 10.5465/annals.2016.0095

[B97] Strack F. Deutsch R. (2004). Reflective and impulsive determinants of social behavior. Pers. Soc. Psychol. Rev. 8, 220–247. 10.1207/s15327957pspr0803_1 15454347

[B98] Szrodecki K. (2018). Obesity discrimination destroys my career. BBC News. Available online at: https://www.bbc.co.uk/news/av/health-45825719/obesitydiscrimination-damaged-my-career (accessed April 20, 2020).

[B99] Taber C. S. Lodge M. (2006). Motivated skepticism in the evaluation of political beliefs. Am. J. Pol. Sci. 50, 755–769. 10.1111/j.1540-5907.2006.00214.x

[B100] Taghipour A. Dejban R. (2013). Job performance: mediate mechanism of work motivation. Proc. Soc. Behav. Sci. 84, 1601–1605. 10.1016/j.sbspro.2013.06.796

[B101] Tangen S. (2003). An overview of frequently used performance measures. Work Stud. 52, 347–354. 10.1108/00438020310502651

[B102] Täuber S. Mulder L. B. Flint S. W. (2018). The impact of workplace health promotion programs emphasizing individual responsibility on weight stigma and discrimination. Front. Psychol. 9:2206. 10.3389/fpsyg.2018.02206 30510529 PMC6253158

[B103] Tepper B. J. Moss S. E. Duffy M. K. (2011). Predictors of abusive supervision: supervisor perceptions of deep-level dissimilarity, relationship conflict, and subordinate performance. Acad. Manag. J. 54, 279–294. 10.5465/amj.2011.60263085

[B104] Thomas D. M. Martin C. K. Heymsfield S. Redman L. M. Schoeller D. A. Levine J. A. (2011). A simple model predicting individual weight change in humans. J. Biolog. Dyn. 5, 579–599. 10.1080/17513758.2010.508541 24707319 PMC3975626

[B105] Tovey M. Herdman C. M. (2014). Seeing changes: how familiarity alters our perception of change. Vis. Cogn. 22, 214–238, 10.1080/13506285.2014.89416727242627

[B106] Tversky A. Kahneman D. (1974). Judgment under uncertainty: heuristics and biases. Science 185, 1124–1131. 10.1126/science.185.4157.1124 17835457

[B107] Van der Zee R. (2017). Demoted or dismissed because of your weight? the reality of the size ceiling. The Guardian. Available online at: https://www.theguardian.com/inequality/2017/aug/30/demoted-dismissed-weight-size-ceiling-workdiscrimination (accessed April 20, 2020).

[B108] Vierck E. Kiesel A. (2008). Change detection: evidence for information accumulation in flicker paradigms. Acta Psychol. 127, 309–323. 10.1016/j.actpsy.2007.06.004 17662952

[B109] Wang M. (2007). Profiling retirees in the retirement transition and adjustment process: examining the longitudinal change patterns of retirees' psychological well-being. J. Appl. Psychol. 92, 455–474. 10.1037/0021-9010.92.2.455 17371091

[B110] Wang M. Burlacu G. Truxillo D. James K. Yao X. (2015). Age differences in feedback reactions: the roles of employee feedback orientation on social awareness and utility. J. Appl. Psychol. 100, 1296–1308. 10.1037/a0038334 25546265

[B111] WHO (2004). Public health Appropriate body-mass index for Asian populations and its implications for policy and intervention strategies. Lancet 363, 157–163. 10.1016/S0140-6736(03)15268-3 14726171

[B112] Woehr D. J. (1994). Understanding frame-of-reference training: the impact of training on the recall of performance information. J. Appl. Psyhcol. 79, 525–534. 10.1037/0021-9010.79.4.525

